# Supplementary material for: School Health: Pediatric Primary Care Curriculum
Source: MedEdPORTAL. 2018 Oct 19;14:10764. doi: 10.15766/mep_2374-8265.10764 (PMC6346276; doi:10.15766/mep_2374-8265.10764)
Supplement: Supplementary file 1 — A. School Health Curriculum Preparation Checklist.docx B. Part 1 Lession Plan.docx C. School Health Didactic Series Presurvey.docx D. School Accommodations Pre Posttest.docx E. Comparison Table.docx F. Part 2 Lesson Plan.docx G. Role-Play.docx H. Part 3 Lesson Plan.docx I. School Personnel Pre Posttest Answer Key.docx J. Responsibilities of School Health Aide and School Nurse.docx K. Medication Administration Form Instructions.docx L. Assignments.docx M. Follow-up Session.docx N. School Health Didactic Series Postsurvey.docx [file mep-14-10764-s001.zip › A._School_Health_Curriculum_Preparation_Checklist.docx]

***Beginning of the Academic Year***

Schedule dates for didactic sessions – 1 hour is needed per part (1-3)

Schedule dates for follow-up session (ideally 4-6 weeks after didactic sessions)

Identify facilitators

Family navigator to be present for Part 2

*At our institution, a family navigator is a registered nurse who assists families with referrals, forms and other logistics of obtaining community resources. At your institution, this may be a social worker, advanced care practitioner, etc., and they may have a different label.*

Parents of patients with special needs (ideally 2-3) to be present for Part 2

*To identify these families, we reached out to the family navigators in primary care and in behavior and development clinics. We invited older patients to join as well. Consider hospital- or community-sponsored support groups as another means to find participants.*

School nurse(s) to be present for Part 3

*To identify these nurses, ask if your hospital has a school nurse council or simply reach out to schools in the community.*

***2 Weeks Before a Didactic Session***

Send out reminder emails to facilitators

Confirm room reservations

***1 Week Before a Didactic Session***

Send out reminder email to resident participants

Print handouts or make electronically available to participants:

- Part 1 – School Accommodations
  - School Accommodations Pre/Post-tests & Answer Key (*Appendix C)*
  - Comparison Table of IHP, IEP & 504 *(Appendix D)*
  - Examples of IEPs, 504s & IHPs *(see Appendix B References)*
- Part 2 – IEP Process
  - Role play scenarios & observer checklist (*Appendix F*)
- Part 3 – School Staff and Communication
  - School Personnel Pre/Post-tests & Answer Key (*Appendix H*)
  - Comparison Table: School Health Aide vs School Nurse *(Appendix I)*
  - Medication Administration Form Instructions (*Appendix J*)
  - Example medication administration form *(see Appendix G References)*
- Follow-up assignments (*Appendix K*) – to be distributed after Parts 1-3 are completed

***Day of the Didactic Session***

Send email with assignment descriptions (*Appendix K*)

***1 Week Before the Follow-up Session***

Send out reminder email to participants, include list of assignments
