# Supplementary material for: School Health: Pediatric Primary Care Curriculum
Source: MedEdPORTAL. 2018 Oct 19;14:10764. doi: 10.15766/mep_2374-8265.10764 (PMC6346276; doi:10.15766/mep_2374-8265.10764)
Supplement: Supplementary file 1 — A. School Health Curriculum Preparation Checklist.docx B. Part 1 Lession Plan.docx C. School Health Didactic Series Presurvey.docx D. School Accommodations Pre Posttest.docx E. Comparison Table.docx F. Part 2 Lesson Plan.docx G. Role-Play.docx H. Part 3 Lesson Plan.docx I. School Personnel Pre Posttest Answer Key.docx J. Responsibilities of School Health Aide and School Nurse.docx K. Medication Administration Form Instructions.docx L. Assignments.docx M. Follow-up Session.docx N. School Health Didactic Series Postsurvey.docx [file mep-14-10764-s001.zip › B._Part_1_Lession_Plan.docx]

**School Health Curriculum**

**Part 1 Lesson Plan – School Accommodations**

**(1 hour)**

**Learning Objectives**

1. Articulate the differences between an Individualized Educational Plan (IEP), Individualized Health Plan (IHP) and 504 plan, as assessed by a pre- and post-didactic survey.
2. Identify clinical scenarios in which a patient would qualify for an IEP, IHP and/or 504 plan, as assessed by a pre- and post-didactic survey.

**Materials/Personnel**

- Facilitators: faculty/resident curriculum leaders
- School Health Didactic Series Pre-Survey (*Appendix C*)
- School Accommodations Pre/Post-tests & Answer Key (*Appendix D)*
- Comparison Table of IHP, IEP & 504 *(Appendix E)*
- Examples of IEPs, 504s & IHPs *(see References below)*

**Introduction (10 minutes)**

1. Introductions – ask about any prior experience with school health
2. Brief overview of the session
   1. Emphasize that it is important for pediatricians to be aware of these plans, but that they are not expected to be experts. Schools have full authority over what is included in an accommodation plan (i.e. IEP or 504) and school nurses are the experts on how to provide medical care in the school environment. Pediatricians should encourage families to apply for needed accommodations but should be respectful of the school’s authority.
   2. The actual layout of these plans vary by state and school district, but the general scope is universal.
3. Fill out pre-series survey and pre-tests (*Appendix C, Appendix D)*

**Individualized Education Program (IEP) (14 minutes)**

1. *Definition:* a plan protected under the Individuals with Disabilities Education Act (IDEA) to ensure specialized education and services for disabled children in elementary and secondary schools.
2. *Who qualifies?*
   1. Children with disabilities that impact their educational performance and/or their ability to learn. They must have a diagnosis that aligns with one of the categories of disability under the IDEA (*see Appendix E*).
   2. *Example:* A student with cerebral palsy is provided with physical and occupational therapy during school.
3. *Who creates it?* A multidisciplinary team, which includes a general education teacher, a special education teacher, a school psychologist, a school district representative and ideally a parent, must meet to construct a student’s IEP. The role of the physician is to identify health conditions that may affect access to education. The physician is rarely involved in the development of the IEP plan but essentially serves as a resource for health concerns.
4. *What is included in the plan?* While the IEP may vary by school district, the key components include: (1) the date of the initial IEP, (2) date(s) of subsequent reviews, (3) the student’s identified disabilities, (4) the student’s goals, (5) the accommodations or modifications to be provided and (6) specific services to be delivered at school, including physical, occupational and speech therapies.
5. Review a sample plan. *See References.*

**504 Plan (14 minutes)**

1. *Definition:* a plan to provide accommodations for disabled children in elementary and secondary school to ensure access to learning and academic success.
2. *Who qualifies?*
   1. Children with disabilities that impact their ability to learn in a general education class.
   2. *Example:* A student with ADHD is given extended time on all tests.
3. *Who creates it?* A multidisciplinary team who is familiar with the student, which may include a teacher, school administrator and parent. The physician role is to screen, diagnose and refer to resources. Physicians are rarely involved in the development of the 504 plan but may be needed to provide a medical diagnosis.
4. *What is included in the plan?* Again, 504 plans will vary by school district and state, but will include information on: (1) date of the plan, (2) the student’s disability and (3) a list of accommodations.
5. Review a sample plan. *See References.*

**Individualized Health Plan (IHP) (14 minutes)**

1. *Definition:* a care plan to address healthcare conditions at school that may impact school performance and/or attendance.
2. *Who qualifies?*
   1. Children with medical problems that require modification of the school environment, special diets, in-school medication administration, assistance with self-care or special training of school staff.
   2. *Example:* A child with exercise-induced asthma should have access to their rescue inhaler at school and should have a plan to use albuterol prior to gym class.
3. *Who creates it?* The school nurse, ideally in conjunction with the student’s family and healthcare provider.
4. *What is included in the plan?* IHPs have the greatest variability. Reach out to a local school nurse for more information.
5. Review a sample plan. *See References.*

**Wrap-up (8 minutes)**

1. Fill out post-tests (*Appendix D*)
2. Review correct answers to school accommodations scenarios
   1. Split into small groups for discussion of answers, 1 facilitator per group
   2. Compare pre-test to post-test answers
3. Questions

**References**

- Sample IEP:
  IEP Example. *Special Education Advisor.* Available at: <http://www.specialeducationadvisor.com/iep-example/>. Accessed August 13, 2018.
- Sample 504 plan:
  Section 504 Plan for ADHD. *Understood.* Available at: https://www.understood.org/~/media/231736e1178c4708b09749acc1e6e3b8.pdf. Accessed August 13, 2018.
- Sample IHP:
  Individual health care plans and emergency information forms. *Pacer Center.* Available at: <http://www.pacer.org/health/samplehealthplans.asp>. Accessed August 5, 2016.
- Further Reading:
  - American Academy of Pediatrics Committee on Children with Disabilities. The Peditrician’s role in development and implementation of an Individualized Education Plan (IEP) and/or an Individual Family Service Plan. *Pediatrics*. 1999;104(1): 124-27.
  - Anatomy of an IEP. *Understood.* Available at: <https://www.understood.org/en/school-learning/special-services/ieps/at-a-glance-anatomy-of-an-iep>. Accessed August 13, 2018.
  - Contents of the IEP. *Center for Parent Information & Resources.* Available at: <https://www.parentcenterhub.org/iepcontents/#contents>. Accessed August 20, 2018.
  - Sampson CH, Galemore CA. What Every School Nurse Needs to Know
     About Section 504 Eligibility. *NASN School Nurse.* 2012;27(2): 88-93.
  - About Specific Disabilities. Center for Parent Information and Resources. Web
     site: <http://www.parentcenterhub.org/specific-disabilities/>. Accessed July 14,
     2017.
  - Individualized Healthcare Plan Process. *Colorado Department of Education*; 2010.
     Available at: <https://www.cde.state.co.us/sites/default/files/documents/healthandwellness/download/nurhealthcareplanprocess.pdf>. Accessed January 4, 2018.
