# Supplementary material for: School Health: Pediatric Primary Care Curriculum
Source: MedEdPORTAL. 2018 Oct 19;14:10764. doi: 10.15766/mep_2374-8265.10764 (PMC6346276; doi:10.15766/mep_2374-8265.10764)
Supplement: Supplementary file 1 — A. School Health Curriculum Preparation Checklist.docx B. Part 1 Lession Plan.docx C. School Health Didactic Series Presurvey.docx D. School Accommodations Pre Posttest.docx E. Comparison Table.docx F. Part 2 Lesson Plan.docx G. Role-Play.docx H. Part 3 Lesson Plan.docx I. School Personnel Pre Posttest Answer Key.docx J. Responsibilities of School Health Aide and School Nurse.docx K. Medication Administration Form Instructions.docx L. Assignments.docx M. Follow-up Session.docx N. School Health Didactic Series Postsurvey.docx [file mep-14-10764-s001.zip › C._School_Health_Didactic_Series_Presurvey.docx]

School Health Didactic Series Pre-Survey

*Please write the last 4 digits of your phone number in the top right corner of every page to help us track results. We promise we won’t look you up!*

**DEMOGRAPHICS**

Age: ____

Gender: Male Female

Year of Training: PL-1 PL-2

Location of continuity clinic:

Work experience prior to medical school: ________________________________ or N/A

Undergraduate major: ________________________________________________

Career plans: Primary Care Hospitalist Specialist Undecided

**SH BACKGROUND**

What exposure have you had on school health before now?

1. A lot
2. Some
3. None

Please indicate what training, if any, you have had about school health during **medical school.**

1. Discussion with an attending or health care team member when seeing a patient in clinic.
2. Small group lecture in clinic
3. Reading from a journal article or textbook
4. Half day of clinic at a school-based health center
5. None
6. Other: _______

Please indicate what training, if any, you have had about school health so far in **residency**.

1. Discussion with an attending or health care team member when seeing a patient in clinic.
2. Small group lecture in clinic
3. Reading from a journal article or textbook
4. Half day of clinic at a school-based health center
5. None
6. Other: _______

**SH KNOWLEDGE**

How confident are you that you have the knowledge to help a family of child with special health needs obtain necessary school services?

1. Not at all
2. Slightly
3. Somewhat
4. Very

**SH BEHAVIORS**

Have you ever explained to families in clinic about accommodations (i.e. IEP, 504 plan, IHP) for students with known physical or intellectual disabilities?

1. Yes; for how many families? ___
2. No

How comfortable do you feel in explaining to a family the accommodations that may be available to their child and how to pursue those accommodations at school?

1. Not at all
2. Slightly
3. Somewhat
4. Very

Have you ever asked a patient to bring their IEP to primary care clinic for you to review?

1. Yes, and I reviewed it
2. Yes, but I didn’t look at it
3. Yes, but the patient forgot to bring it
4. No

During residency, for how many patients have you contacted a school on their behalf?

1. None
2. 1-4
3. 5-15
4. More than 15

How comfortable do you feel in contacting a school on behalf of a patient?

1. Not at all
2. Slightly
3. Somewhat
4. Very

How much of a barrier to contacting a school are each of the following barriers? Please rank 1-3 (1 = biggest barrier; 3= smallest barrier)

___ I don’t know who to contact.

___ I don’t feel comfortable.

___ I don’t have time.

___ Other: _____________________________________________________________________

How frequently do you ask patients about medications they may be taking at school?

1. Never
2. Only at well child checks
3. Only at visits resulting in a new medical diagnosis
4. Only at visits resulting in a new medical diagnosis or well child checks
5. At each visit

If you answered B-E to the above question, how often do you provide a medication administration form?

1. Never
2. Only at well child checks
3. Only at visits result in a new medical diagnosis
4. Only at visits resulting in a new medical diagnosis or well child checks
5. At each visit

How often do you provide an “action plan” for a patient’s medical condition and recommend they take it to school?

1. Never
2. Only at well child checks
3. Only at visits resulting in a new medical diagnosis
4. Only at visits resulting in a new medical diagnosis or well child checks
5. At each visit

If you do provide “action plans” (i.e. answered B-E to the last question), for which diagnoses do you do so? _____________________________________________________________________
