# Supplementary material for: School Health: Pediatric Primary Care Curriculum
Source: MedEdPORTAL. 2018 Oct 19;14:10764. doi: 10.15766/mep_2374-8265.10764 (PMC6346276; doi:10.15766/mep_2374-8265.10764)
Supplement: Supplementary file 1 — A. School Health Curriculum Preparation Checklist.docx B. Part 1 Lession Plan.docx C. School Health Didactic Series Presurvey.docx D. School Accommodations Pre Posttest.docx E. Comparison Table.docx F. Part 2 Lesson Plan.docx G. Role-Play.docx H. Part 3 Lesson Plan.docx I. School Personnel Pre Posttest Answer Key.docx J. Responsibilities of School Health Aide and School Nurse.docx K. Medication Administration Form Instructions.docx L. Assignments.docx M. Follow-up Session.docx N. School Health Didactic Series Postsurvey.docx [file mep-14-10764-s001.zip › D._School_Accommodations_Pre_Posttest.docx]

School Accommodations Pre/Post-Test

Define the following:

1. IEP: ____________________________________________________________________
2. 504: ____________________________________________________________________
3. IHP: ____________________________________________________________________

*In each of the following examples, does the student need an IEP, 504 or IHP? You may give multiple answers. Please describe in a few words why you chose the specific intervention.*

1. Jane is a 4-year-old girl with significant expressive language delay who moved from New Mexico to Colorado 6 months ago. She was receiving services through early intervention but was in the process of getting evaluated by another program. Since moving to Colorado, she has not received any speech or related services.
2. Becky, a 16-year-old girl with severe allergies to ferrets and severe persistent asthma, is seen by you in adolescent clinic. She is complaining that there is a ferret in the classroom right next to where she sits. She also gets very short of breath participating in some of the activities in PE, despite using albuterol.
3. Sam is an 11-year-old boy with moderate ADHD, who is often distracted during class, gets out of his seat without permission and is unable to complete tests in the time given. However, he is currently passing all classes.
4. Josh, an 11-year-old with severe ADHD and ODD resulting in several suspensions in the last couple of years, sees you in continuity clinic for behavioral concerns. He has been getting D’s and F’s due to incomplete assignments, frequent disruptions in the classroom, and teacher defiance.
5. Marissa is a 13-year-old with juvenile idiopathic arthritis who have has significant stiffness and limited range of motion, frequent absences due to pain and multiple medication needs.
6. Caleb is a 10-year-old with newly diagnosed type I diabetes who is prescribed a sliding scale and carbohydrate correction insulin regimen for all meals.

School Accommodations Scenario Answer Key

Definitions – answers should include bolded concepts to receive credit.

1. IEP – a plan protected under the Individuals with Disabilities Education Act (IDEA) to **ensure specialized education and services** for disabled children in elementary and secondary schools. *Correct answers could include comments about placement in a special education program and/or therapies (such as physical therapy, occupational therapy and speech therapy) as well as the idea that the school program is catered to the strengths and weakness of the individual student.*
2. 504 – a plan to provide accommodations for disabled children in elementary and secondary school to ensure **access to learning and academic success**. *Correct answers could include comments about adaptations to a general education classroom and extracurricular activities.*
3. IHP – a care plan to **address healthcare conditions** at school that may impact school performance and/or attendance. *Correct answers could include comments about in-school medical interventions, such as action plans and medication administration, that require healthcare provider authorization.*

Case Scenarios:

1. **IEP.** *She may have a learning disability and should get an evaluation and all needed therapies (i.e. physical therapy, occupational therapy, speech therapy) through school*.
2. **504 & IHP**. *She should get accommodations in gym class (i.e. longer time to complete activities, more frequent breaks) and seating far away from animals in the classroom (all covered by a 504). She also needs an asthma action plan and anaphylaxis plan (included in an IHP).*
3. **504.** *He should get accommodations to take in account his ADHD: longer time for testing, preferential seating, stress ball when he’s distracted, etc. Since his ADHD is mild and he is passing his classes, he does not need an IEP.*
4. **IEP.** *He needs an IEP because he is failing his classes and has severe disability from his ADHD and ODD. He needs a full evaluation for learning disability and would likely benefit from a smaller classroom and one-on-one assistance.*
5. **IEP & IHP.** *She needs an IEP because her juvenile idiopathic arthritis causes severe disability. She may need occupational or physical therapy in school and would benefit from having a modified set of goals given her frequent absences. An IHP would help to assist in pain management while she is in the classroom.*
6. **504 & IHP.** *He should be given accommodations that allow him to carry a water bottle, drink juice for symptomatic hypoglycemia, etc. An IHP is needed so that he can check his glucose and ketones and get his insulin at school.*
