# Supplementary material for: School Health: Pediatric Primary Care Curriculum
Source: MedEdPORTAL. 2018 Oct 19;14:10764. doi: 10.15766/mep_2374-8265.10764 (PMC6346276; doi:10.15766/mep_2374-8265.10764)
Supplement: Supplementary file 1 — A. School Health Curriculum Preparation Checklist.docx B. Part 1 Lession Plan.docx C. School Health Didactic Series Presurvey.docx D. School Accommodations Pre Posttest.docx E. Comparison Table.docx F. Part 2 Lesson Plan.docx G. Role-Play.docx H. Part 3 Lesson Plan.docx I. School Personnel Pre Posttest Answer Key.docx J. Responsibilities of School Health Aide and School Nurse.docx K. Medication Administration Form Instructions.docx L. Assignments.docx M. Follow-up Session.docx N. School Health Didactic Series Postsurvey.docx [file mep-14-10764-s001.zip › E._Comparison_Table.docx]

|  | **IHP** | **IEP** | **504** |
| --- | --- | --- | --- |
| **Basic Description** | **Individualized Healthcare Plan** **(IHP)** is a plan for “students whose healthcare needs affect or have the potential to affect safe and optimal school attendance and academic performance” (NASN, 2015). | The **Individualized Educational Plan** **(IEP)** is a plan or program developed to ensure that a child who has a disability identified under the law and is attending an elementary or secondary educational institution receives specialized instruction and related services. | The **504 Plan** is a plan developed to ensure that a child who has a disability identified under the law and is attending an elementary or secondary educational institution receives accommodations that will ensure their academic success and access to the learning environment. |
| **What It Does** | IHP is a legal document showing proof that the school nurse provided a minimum standard of care for a student with a health condition. | Provides individualized special education and related services to meet the unique needs of the child. | Provides services and changes to the learning environment to meet the needs of the child as adequately as other students. |
| **What Law Applies** | **State Specific Nurse Practice Act** | **The Individuals with Disabilities Education Act (IDEA)** is a federal special education law for children with disabilities. https://sites.ed.gov/idea/  State laws can’t contradict IDEA, and they can’t provide *less* than the federal law requires. | **Section 504 of the Rehabilitation Act of 1973 & Americans with Disabilities Act Amendments Act of 2008** is a federal civil rights law to stop discrimination against people with disabilities. <https://www.eeoc.gov/laws/statutes/adaaa.cfm> |
| **Who Is Eligible** | Children with health problems that require:   - Special training of school personnel - Modification in school environment - Added safety measures - Measures to relieve pain - Self-care assistance - Rehabilitation measures - Treatment orders for special procedures - Special diet - Medications or interventions for emergency treatment | Child’s disability affects the child’s educational performance and/or ability to learn and benefit from the general education curriculum  Child has one or more of the Categories of Disability Under IDEA <http://www.parentcenterhub.org/repository/categories/>   - Autism - Deaf-blindness - Deafness - Emotional disturbance - Hearing impairment - Intellectual disability - Multiple disabilities - Orthopedic impairment - Other health impairment - Specific learning disability - Speech or language impairment - Traumatic brain injury - Visual impairment, including blindness | Disability interferes with the child’s ability to learn in a general education classroom and includes the following:   - seeing - hearing - speaking - walking - breathing - performing manual tasks - learning - caring for oneself - working - reading - concentrating - thinking - sleeping - eating   and major bodily functions:   - immune system operation - normal cell growth - digestive - bowel - bladder - neurological - brain - respiratory - circulatory - endocrine - Reproductive functions. |
| **Who Creates the Program/Plan** | The IHP is developed by the school nurse using the nursing process in collaboration with the student, family and healthcare providers. The school nurse utilizes the IHP to provide care coordination, to facilitate the management of the student’s health condition in the school setting, to inform school-educational plans, and to promote academic success. The ECP, written by the school nurse, is for support staff with an individual plan for emergency care for the student. (NASN, 2015) | An IEP is created by a team that must include:   - The child’s parent - At least one of the child’s general education teachers - At least one special education teacher - School psychologist or other specialist who can interpret evaluation results - A district representative with authority over special education services   With a few exceptions, the entire team must be present for IEP meetings. | A 504 plan is created by a team of people who are familiar with the child and who understand the evaluation data and special services options. This might include:   - The child’s parent - General and special education teachers - The school principal |
| **What's in the Program/Plan** | The IHP incorporates and documents the nursing process in student care in accordance with state nurse practice acts. The nursing process provides a framework for the nurse’s responsibility and accountability. The IHP clarifies clinical practice, provides administrative information , and serves as the foundation for health portions of other educational plans and the Emergency Care Plan (ECP) | The IEP is a written document that sets learning goals for a child and describes the services the school provides.   - Students strengths, preferences, interests - Summary of student present level of educational performance - Age appropriate transition assessment process used to develop the post-school goals*.* - Student Needs and impact of disability - Parent/Student input - Measurable post-school goals - Consideration of special factors - Annual goals - Accommodations & Modifications | There is no standard 504 plan. A 504 plan generally includes the following:   - Specific accommodations, supports or services for the child - Names of who will provide each service - Name of the person responsible for ensuring the plan is implemented |
| **Parent Notice** | School nurses should be included in the registration process or other processes where parents inform the school of their child’s health needs. | IDEA 2004 regulations explicitly state that the 60 calendar day time frame for conducting an initial evaluation begins when the public agency receives the parental consent for evaluation.  Prior written notice is required for any IEP meetings and evaluations, or when the school wants to change a child’s services or placement. | Section 504 does not include a clearly established "Prior Written Notice" requirement. Does not include these protections. |
| **Parent Consent** | Parents provide written consent to implement an IHP that contains medication or therapeutic interventions prescribed by a licensed health care provider. The school nurse may implement a safety and monitoring IHP for students without parent or provider authorization. | A parent must consent in writing for the school to evaluate a child and before the school can provide services in an IEP. | A parent’s consent is required for the school district to evaluate a child. |
| **How Often It’s Reviewed and Revised** | The IHP should be reviewed at least annually and as indicated by changes in student’s health status. | The IEP team must review the IEP at least once a year.  The student must be reevaluated every three years to determine whether services are still needed. | There is no true statutory time line that schools must abide by with regard to updating 504 Plans (unlike IEPs, which must be updated annually). The regulations simply say that the plan should be revisited periodically. (Wright, 2008) |
| **How to Resolve Disputes** | As a care coordinator the nurse is in a key role to facilitate the resolution of disputes. | IDEA requires school districts to conduct impartial hearings for parents who disagree with identification, evaluation, or placement. | Section 504 requires school districts to conduct impartial hearings for parents who disagree with identification, evaluation, or placement. The parent has an opportunity to participate and obtain representation by counsel, but other details are left to the discretion of the school district. |
| **Funding/Costs** | Students receive IHP services at no charge. | Students receive IEP services at no charge.  If the IEP indicates that the child needs nursing services per the Individualized Healthcare Plan and this child is also covered under Medicaid, these direct care services may be reimbursable under the state’s Medicaid plan | Students receive 504 services at no charge.  States do not receive extra funding for eligible students. The federal government can take funding away from programs (including schools) that don’t comply. |

References

Galemore, C. A., & Sheetz, A. H. (2015). IEP, IHP, and Section 504 Primer for New School Nurses. *NASN School Nurse*, 85-88.

National Association of School Nurses. (2015, January). *Position Statement: Individualized healthcare plans.* Retrieved June 15, 2016, from NASN: https://www.nasn.org/nasn/advocacy/professional-practice-documents/position-statements/ps-ihps

National Association of School Nurses. (2018, January). *Position Statement: Reimbursement for School Nursing Healthcare Services.* Retrieved June 2016, from NASN: https://www.nasn.org/nasn/advocacy/professional-practice-documents/position-statements/ps-reimbursement

Nii, P. B., & Baker, C. (2018, January). *IEP-IHP-504 table.* Retrieved from School Health Program Education Team SharePoint: www.childrenscolorado.org

Wright, P., & Wright, P. (2008, March). *Key Differences Between Section 504, the ADA, and the IDEA*. Retrieved June 2016, from Wrightslaw: http://www.wrightslaw.com/info/sec504.summ.rights.htm
