# Supplementary material for: School Health: Pediatric Primary Care Curriculum
Source: MedEdPORTAL. 2018 Oct 19;14:10764. doi: 10.15766/mep_2374-8265.10764 (PMC6346276; doi:10.15766/mep_2374-8265.10764)
Supplement: Supplementary file 1 — A. School Health Curriculum Preparation Checklist.docx B. Part 1 Lession Plan.docx C. School Health Didactic Series Presurvey.docx D. School Accommodations Pre Posttest.docx E. Comparison Table.docx F. Part 2 Lesson Plan.docx G. Role-Play.docx H. Part 3 Lesson Plan.docx I. School Personnel Pre Posttest Answer Key.docx J. Responsibilities of School Health Aide and School Nurse.docx K. Medication Administration Form Instructions.docx L. Assignments.docx M. Follow-up Session.docx N. School Health Didactic Series Postsurvey.docx [file mep-14-10764-s001.zip › F._Part_2_Lesson_Plan.docx]

**School Health Curriculum**

**Part 2 Lesson Plan – IEP Process**

**(1 hour)**

**Learning Objectives**

1. Explain an IEP, IHP or 504 plan to a family so that they feel confident in advocating for their children’s needs to optimize their education within schools, as assessed by graded role plays.
2. Explain to a patient’s family how to request an evaluation for an IEP so a family feels empowered to advocate for their child’s school needs, as assessed by graded role plays.

**Materials/Personnel**

- Facilitators: faculty/resident curriculum leaders, family navigator, 2-3 parents of patients with special needs *(see Appendix A for more information on identifying these facilitators)*
- Role play scenarios & observer checklist (*Appendix G*)

**Introduction (5 minutes)**

1. Introductions – explain unique background of session facilitators
2. Brief overview of the session

**Process of Obtaining an IEP (25 minutes)**

1. Large group discussion, led by the family navigator.
2. Sample questions to discuss:
   - 1. What does the process for obtaining an IEP look like?

*The basic steps are: (1) the parents ask for an IEP via written request; (2) the parents bring the request letter to the school and have the secretary sign and date the document for the school, leaving one copy for the family’s records; (3) the school determines a plan for evaluation; (4) evaluation occurs; and (5) the IEP meeting is held to discuss results of the evaluation and plans for any accommodations.*

- - 1. What should be included in the written request from the family?

*The letter should include child’s name, date of birth, a named teacher or class in which the child could benefit from the support of an IEP.*

- - 1. What is a school’s legal obligation in evaluating a student?

*If the parent asks for an evaluation in writing, the school is legally bound to evaluate the student within 60 days. If the school says the child will not qualify, the parent needs to say, “we will discuss eligibility after all of the testing, evaluations and observations are completed.” The school needs to have an eligibility meeting with the parent to discuss results, regardless if the child is eligible for services, to outline how the school arrived at the results, indicating test measures. Email or verbal request does not constitute a legal response from the school to evaluate.*

- - 1. What testing and what diagnoses can a school make?

*Schools can test for learning disabilities, including dyslexia, autism and IQ. However, they would be considered educational diagnoses, not medical. Testing will vary by school, but may include a combination of observations, interviews, psychometric tests (e.g. Wechsler Individual Achievement Test) and curriculum-based assignments. See “References” below for more information on psychometric testing.*

- - 1. How long does this process usually take?

*The school is required to have the testing completed within 60 days (not including weekends and holidays). The district should provide the parent a plan for assessment and a copy of the procedural safeguards within 15 days of receiving the referral.*

- - 1. Who needs to be in an IEP meeting?

*Attendees should include at least one of the child’s general education teachers, someone who can interpret results (i.e., school psychologist), the parents, a school system representative (from the special education department), a transition service agency if the child is of transition age (i.e. in high school) and the student. Note, however, that neither the parents nor the student must be there for the meeting to take place.*

- - 1. If we recommend to a family that a child should get an IEP, what can we do as pediatricians to expedite or facilitate the process?

*Physicians cannot expedite the process with the school nor legally recommend any accommodations for the child at school. The school district needs to do their own testing, evaluations and observations. Physicians can explain the process to parents, as described above, and provide documentation of medical diagnoses that the school might consider via record review.*

- - 1. What are common challenges that families typically encounter during this process?

*Families often do not know their rights related to what their child is legally able to access via an IEP. There are local and federal organizations (with websites) to whom you can refer your families to help educate them. See “Online Family Resources” in References below.*

- - 1. How do you usually communicate the need for an IEP to a patient’s family?

*A physician can encourage the parent to have the child tested as described above if the parent suspects that the child would benefit from additional supports at school.*

**Family Panel to discuss experiences in obtaining IEP (15 minutes)**

1. Large group Q&A, to discuss family experience in obtaining IEPs.
   1. Given the limited time, encourage residents to focus their questions on the patient/family experience.
2. Ask each family member to provide a short introduction to their child and experience in obtaining IEPs.
3. Sample questions to discuss
   1. What went well and what was frustrating about your IEP experience?
   2. What did you wish you knew beforehand?
   3. How do you think your pediatrician could help in this process?

**Activity: Role play (10 minutes)**

1. Hand out role play scenarios & observer checklist (*Appendix G)*
2. Divide into small groups, with at least 2 residents and 1 parent or family navigator per group
   1. Residents should play the role of the pediatrician and observer.
   2. Parent or family navigator should play the role of parent in both scenarios but should also give feedback to the residents on their communication.
3. Each small group may select their scenario. The alternant scenario should be used if time permits.

**Wrap-up (5 minutes)**

1. Final words of wisdom from parents and family navigator
2. Questions

**References**

- A Guide to the Individualized Education Program. *Office of Special Education and
   Rehabilitative Services U.S. Department of Education*; 2000. Available at:
   <https://www2.ed.gov/parents/needs/speced/iepguide/iepguide.pdf>. Accessed
   January 4, 2018.
- Evaluating Children for Disability. *Center for Parent Information & Resources*. Available at: <https://www.parentcenterhub.org/evaluation/#scope>. Accessed August 20, 2018.
- IEP Preparation Packet. *PEAK Parent Center*; 2008. Available at:
  <https://www.peakparent.org/sites/default/files/peak_parent_center_iep_prep_packet_english.pdf>. Accessed August 5, 2016.
- Psychometric Testing. *Medical Home Portal.* Available at: <https://www.medicalhomeportal.org/issue/psychometric-testing>. Accessed August 20, 2018.
- Online Family Resources
  - Center for Parent Information and Resources. Website: <http://www.parentcenterhub.org/>
  - PEAK Parent Center. Website: <https://www.peakparent.org/>
  - Understood. Website: <https://www.understood.org/>
  - Wright’s Law. Website: <http://www.wrightslaw.com/>
