# Supplementary material for: School Health: Pediatric Primary Care Curriculum
Source: MedEdPORTAL. 2018 Oct 19;14:10764. doi: 10.15766/mep_2374-8265.10764 (PMC6346276; doi:10.15766/mep_2374-8265.10764)
Supplement: Supplementary file 1 — A. School Health Curriculum Preparation Checklist.docx B. Part 1 Lession Plan.docx C. School Health Didactic Series Presurvey.docx D. School Accommodations Pre Posttest.docx E. Comparison Table.docx F. Part 2 Lesson Plan.docx G. Role-Play.docx H. Part 3 Lesson Plan.docx I. School Personnel Pre Posttest Answer Key.docx J. Responsibilities of School Health Aide and School Nurse.docx K. Medication Administration Form Instructions.docx L. Assignments.docx M. Follow-up Session.docx N. School Health Didactic Series Postsurvey.docx [file mep-14-10764-s001.zip › G._Role-Play.docx]

Role Play

You will now have the opportunity to practice your communication skills to teach a patient’s family about school accommodations and the process of obtaining an IEP evaluation.  Each scenario should take 10 minutes: 5 minutes to role play and 5 minutes to debrief. You may select from the two scenarios below. If you finish ahead of schedule, please continue to the alternant scenario.

For each scenario, there are 3 roles:

1. Pediatrician (a resident)
2. Patient’s family member (an actual patient’s family member or patient navigator)
3. Observer (the rest of the group)

**SCENARIO 1:**

**Pediatrician:** You are seeing Jon, a patient you know well who has mild autism but has been struggling in school for the last two years. He is currently in the 7th grade. You want to discuss with the parents, who are both at the appointment, about the possibility of obtaining an IEP, how to obtain it and what it will mean for Jon.

**Parent:** You are one of Jon’s parents. Jon has mild autism but has been struggling in school for the last two years. You are frustrated because the medication has not helped his grades and you are not sure what else to do. You are also overwhelmed with three other kids at home and have a busy job. When the pediatrician discusses an IEP, you are apprehensive at first, but then are willing to give it a try, for Jon’s benefit.

**SCENARIO 2:**

**Pediatrician:** You are seeing Rachel, a patient you have seen once before, but is not someone you know very well.

**Patient:** You are the adopted parent of Rachel (adopted a year ago), who is here for her 7-year-old well child check. You are concerned because Rachel has not done well in school this past year and you are worried that she has a learning disability and needs more help in school. You have lots of questions to ask the pediatrician.

Role Play Observer Checklist

During the role play, please evaluate for the items below and provide feedback after the scenario is complete.

Uses patient-friendly language

Explains what an IEP is

Gives examples of things that an IEP can offer that may help this patient

Explains how to obtain an IEP and the expected timeline of events
