# Supplementary material for: School Health: Pediatric Primary Care Curriculum
Source: MedEdPORTAL. 2018 Oct 19;14:10764. doi: 10.15766/mep_2374-8265.10764 (PMC6346276; doi:10.15766/mep_2374-8265.10764)
Supplement: Supplementary file 1 — A. School Health Curriculum Preparation Checklist.docx B. Part 1 Lession Plan.docx C. School Health Didactic Series Presurvey.docx D. School Accommodations Pre Posttest.docx E. Comparison Table.docx F. Part 2 Lesson Plan.docx G. Role-Play.docx H. Part 3 Lesson Plan.docx I. School Personnel Pre Posttest Answer Key.docx J. Responsibilities of School Health Aide and School Nurse.docx K. Medication Administration Form Instructions.docx L. Assignments.docx M. Follow-up Session.docx N. School Health Didactic Series Postsurvey.docx [file mep-14-10764-s001.zip › H._Part_3_Lesson_Plan.docx]

**School Health Curriculum**

**Part 3 Lesson Plan – School Staff and Communication**

**(1 hour)**

**Learning Objectives**

1. Identify the medical services that can and cannot be provided by the school nurse and other school personnel, as assessed by a pre- and post-didactic survey.
2. Develop an individualized health care plan for a patient with a chronic illness through collaboration with the school nurse, as assessed by a medication administration form assignment.

**Materials/Personnel**

- Facilitators: faculty/resident curriculum leaders, school nurse(s) *(see Appendix A for more information on identifying these facilitators)*
- School Personnel Pre/Post-tests & Answer Key (*Appendix I*)
- Comparison Table: Responsibilities of School Health Aide and School Nurse *(Appendix J)*
- Medication Administration Form Instructions (*Appendix K*)
- Example medication administration form *(see References below)*

**Introduction (10 minutes)**

1. Introductions – explain unique background of session facilitators
2. Brief overview of the session
3. Fill out pre-tests (*Appendix I*)

**Overview of school staff (15 minutes)**

1. Large group discussion
2. Provide handout: Responsibilities of School Health Aide and School Nurse (*Appendix J*)
3. Emphasize that nursing services vary greatly by school district and sometimes even by school. Answers to the sample questions below provide general answers, but it is important for providers to be familiar with local affairs. Residents should know the following about their local school district(s): (1) the type of personnel available, (2) the school nurse (or psychologist, etc.) to student ratio and (3) the scope of their role, as dictated by local law.
4. Sample questions to discuss
   1. Who is available to provide care to students at school (i.e. school RN, unlicensed assistive personnel, school psychologist, school counselor)?
      1. *Psychologists, counselors, and social workers working in educational settings usually provide the social, emotional, behavioral health care they are licensed for. These specialized service providers (as they are known in Colorado) seldom provide direct care to students. If direct health care tasks are provided by these professionals, it is usually delegated by the registered nurse (RN).*
      2. *In states where the ratio of students to school nurse is very high, health care tasks are delegated by a RN to unlicensed assistive personnel (UAP). The UAP could be licensed educational staff, including teachers, administrators, counselors, psychologists, or social workers, but is more likely to be paraprofessional staff hired to serve as health aids, secretaries, educational paras who provide health related care tasks after training and delegation by the RN.*
   2. What is in their scope of practice?
      1. How are health care tasks delegated?
         1. *It is helpful for the practicing pediatrician to know the current school nurse-to-student ratios in the state where s/he is practicing. Additionally, if the ratios are poor it would be essential to know if the Licensed Professional Nurse Practice Act includes a delegatory clause. Many states do not have a delegatory clause in their nursing practice act which means the RN is unable to delegate health care tasks to UAPs.*
      2. What medications can they administer?
         1. *Medications that can be administered by unlicensed assistive personnel varies by state according to the Nurse Practice Act. For example, in Colorado, we do not delegate any medications that require nursing judgment to administer like determining whether a child needs a blood pressure specific medication dose. Some states do not allow for UAPs to administer insulin. Colorado may delegate insulin within very specific guidelines described within the rules of the Nurse Practice Act. Even with these guidelines some districts have chosen not to delegate insulin administration and have coordinated their professional nursing resources to manage diabetes care without delegating insulin.*
         2. *Who can administer over-the-counter medications vary widely state to state and within individual districts within each state.*
   3. What is the staffing like at schools in the local school district?

**Communication between Schools and Health Care Providers (15 minutes)**

1. Large group discussion
2. Sample questions to discuss
   - 1. What is FERPA? How does it compare to HIPPA?
        1. *FERPA (Family Educational Rights and Privacy Act) protects student educational records, including health information, school nurse notes and special education records. Under FERPA, parents and students have rights to review and/or amend their records and rights to maintain privacy. FERPA applies to all public and private schools that accept federal funding.*
        2. *HIPAA (Health Insurance Portability and Accountability Act) protects individually identifiable health information (PHI). HIPAA applies to health care providers and health care plans. It only applies to schools when they are billing Medicaid, as is the case with school-based health centers. School health records are outside the scope of HIPAA.*
     2. When should a pediatrician contact a school nurse?
        1. *Ideally the pediatrician would contact the school nurse to find out what the school nursing ratios look like, what the Nurse Practice Act allows and how that translates into health care in the student’s specific school setting.*
        2. *Once the pediatrician is familiar with the health care resources in the school setting, most school nurses will value hearing from the pediatrician about care coordination issues, new diagnoses (e.g. anaphylaxis, diabetes), changes in treatment plans affecting school engagement and support needed for students with chaotic families. Most school nurses will accommodate giving daily medications not usually administered at school (e.g. preventive inhalers) during school hours if the family needs this extra support. Additionally, pediatricians should feel empowered to reach out if there are concerns that physical or mental health issues may be contributing to school attendance or performance.*
        3. *Communication could include a letter, an action plan or a phone call, depending on the complexity of the situation.*
     3. What information is needed from a pediatrician to create an IHP?
        1. *The school nurse will need to know the medical diagnosis, treatment plan, relevant health history and anticipated health care needs and interventions during the school day (which includes transportation to and from school and extracurricular activities).*
        2. *With the above described information, the school nurse will translate the information into a health care plan relevant to the users of the care plan. If the school nurse is the primary user of the IHP, a nursing assessment, nursing diagnoses and outcomes may be included in the IHP. If the users of the plan are UAPs, the assessment and nursing diagnoses are frequently simplified. Assessment, nursing diagnoses, and outcomes are documented elsewhere in the student records.*
     4. What information is needed on a medication administration form to ensure a student is allowed to take medications at school? (*Appendix K*)
        1. *The Right Child. Two identifiers are needed, usually full name and birthdate.*
        2. *The Right Medication. If a name brand is prescribed and the generic is dispensed this can cause a pause in some school settings.*
        3. *The Right Dose. No ranges. Not weight-based. Just the specific dose.*
        4. *The Right Route. Common sense is not always common. If surgery was done on the right eye do you want the medication administered in the right eye?*
        5. *The Right Time. No ranges like 4-6 hours. If it is symptom specific, the symptom must be specific and observable.*
        6. *Purpose of Medication.*
        7. *Side effects that need to be reported.*
   1. Show sample medication administration form *(see References below).*

**Q&A with school RNs (10 minutes)**

1. Large group discussion
2. Sample questions to discuss
   1. How do we identify the nurse working at a particular school?
   2. Is there a way to reach out to nurse to make sure a student with medication noncompliance is taking his/her medicine that was prescribed for school?
   3. What is the biggest limitation to a student receiving medications in school?
   4. If my patient receives a new diagnosis, what is the best way to communicate that to school?

**Wrap-up (10 minutes)**

1. Fill out post-tests (*Appendix I*)
2. Review correct answers to school accommodations scenarios
   1. Split into small groups for discussion of answers, 1 facilitator per group
   2. Compare pre-test to post-test answers
3. Review post-didactic assignments (*Appendix L*)
4. Questions

**References**

- School Staff Definitions:
  - Sterns P. The title ‘nurse’ is now protected in Colorado. Colorado Nurse. 2004;104(3): 10.
  - Unlicensed assistive personnel: their role on the school health services team (Position Statement). National Association of School Nurses; 2015. Available at: <https://www.nasn.org/advocacy/professional-practice-documents/position-statements/ps-uap>. Accessed November 5, 2017.
  - Wilgertodt MA, Brock DM, Maughan EM. Public school nursing practice in the United States. *Journal of School Nursing*. 2018; 34(3), 232-244.
- FERPA vs HIPAA
  - Colorado Department of Education Office of Special Education. Confidentiality and Colorado public school health records. *ESSU Technical Assistance*; 2015: 1-2. Available at: <https://www.cde.state.co.us/cdesped/ta_confidentiality_colopublicschoolhealthrecords>. Accessed August 5, 2016.
  - U.S. Department of Education and U.S. Department of Health and Human Services. Joint Guidance on the Application of the Family Educational Rights and Privacy Act (FERPA) And the Health Insurance Portability and Accountability Act of 1996 (HIPAA) To Student Health Records; 2008: 1-11. Available at: <https://www2.ed.gov/policy/gen/guid/fpco/doc/ferpa-hipaa-guidance.pdf>. Accessed August 5, 2016.
- Individualized Health Care Plans
  - Individualized Healthcare Plans: The Role of the School Nurse. *National Association of School Nurses*. 2015. Available at: <https://www.nasn.org/advocacy/professional-practice-documents/position-statements/ps-ihps>. Accessed August 30, 2018.
  - Individualized Healthcare Plan Process. *Colorado Department of Education*. 2010. Available at: <https://www.cde.state.co.us/sites/default/files/documents/healthandwellness/download/nurhealthcareplanprocess.pdf>. Accessed August 5, 2016.
  - School Nursing and Health – Health Conditions. *Colorado Department of Education*. Web site: <http://www.cde.state.co.us/healthandwellness/snh_healthissues>. Accessed August 5, 2016.
