# Supplementary material for: School Health: Pediatric Primary Care Curriculum
Source: MedEdPORTAL. 2018 Oct 19;14:10764. doi: 10.15766/mep_2374-8265.10764 (PMC6346276; doi:10.15766/mep_2374-8265.10764)
Supplement: Supplementary file 1 — A. School Health Curriculum Preparation Checklist.docx B. Part 1 Lession Plan.docx C. School Health Didactic Series Presurvey.docx D. School Accommodations Pre Posttest.docx E. Comparison Table.docx F. Part 2 Lesson Plan.docx G. Role-Play.docx H. Part 3 Lesson Plan.docx I. School Personnel Pre Posttest Answer Key.docx J. Responsibilities of School Health Aide and School Nurse.docx K. Medication Administration Form Instructions.docx L. Assignments.docx M. Follow-up Session.docx N. School Health Didactic Series Postsurvey.docx [file mep-14-10764-s001.zip › I._School_Personnel_Pre_Posttest_Answer_Key.docx]

School Personnel Pre/Post-Test

*List all school personnel who are legally and professionally capable of completing the following tasks.*

1. Drew is an 8-year-old boy with moderate persistent asthma who comes to the school nurse’s office after PE class for cough and chest tightness without increased WOB. He has an asthma action plan on file. Who can determine where Drew falls on his action plan and provide appropriate treatment?
2. Brayden is a 9-year-old boy with moderate persistent asthma who has poor compliance with medications at home. To help combat poor compliance, his PCP has prescribed his morning Advair to be taken at school at 8am and has provided the school the appropriate documentation to administer the medication. Who can provide his Advair?
3. Sarah, a 12-year-old previously healthy girl, is sent to the school nurse’s office at 9am for feeling poorly after admitting to her teacher that she was up all night with vomiting and diarrhea. She is now complaining of abdominal pain.
   1. Who can take her vital signs?
   2. Who can give her ibuprofen while waiting for a parent to arrive at school?
4. An elementary school in a low-resource neighborhood is performing health screenings on all students. They are measuring BMI and assessing vision and hearing. Who can perform these tasks?

School Personnel Answer Key

1. **School nurse**. Medical training is required to make the assessment of this patient (i.e. to determine where on the asthma action plan he falls). An assistant could provide medication based on subjective symptoms of wheezing or cough, assuming those are written indications on the medical administration form for asthma but could not determine that the child is tachypneic or has increased work of breathing.
2. **Unlicensed assistive personnel (UAP).** An assistant, liked an unlicensed assistive personnel (UAP) or trained secretary, teacher, PE teacher, etc. could administer albuterol presuming they have been trained to do so and there is a current medication administration form on file for this child.
3. (a) **Nurse, CNA, or unlicensed assistive personnel (UAP).** A UAP must be supervised if obtaining vital signs. Note, many school nurses do NOT train their UAPs to do this.

   (b) **Unlicensed assistive personnel (UAP).** The UAP may only provide ibuprofen if the child has a medication administration form for ibuprofen with an indication to give for pain.
4. **Unlicensed assistive personnel (UAP) or anyone trained to complete assessment.** All assistants must be trained to complete these tasks, be aware of the state guidelines for screening and be familiar with the referral process for students with abnormal screenings.
