# Supplementary material for: School Health: Pediatric Primary Care Curriculum
Source: MedEdPORTAL. 2018 Oct 19;14:10764. doi: 10.15766/mep_2374-8265.10764 (PMC6346276; doi:10.15766/mep_2374-8265.10764)
Supplement: Supplementary file 1 — A. School Health Curriculum Preparation Checklist.docx B. Part 1 Lession Plan.docx C. School Health Didactic Series Presurvey.docx D. School Accommodations Pre Posttest.docx E. Comparison Table.docx F. Part 2 Lesson Plan.docx G. Role-Play.docx H. Part 3 Lesson Plan.docx I. School Personnel Pre Posttest Answer Key.docx J. Responsibilities of School Health Aide and School Nurse.docx K. Medication Administration Form Instructions.docx L. Assignments.docx M. Follow-up Session.docx N. School Health Didactic Series Postsurvey.docx [file mep-14-10764-s001.zip › J._Responsibilities_of_School_Health_Aide_and_School_Nurse.docx]

General Questions to Ask

*Specific responsibilities of in-school medical providers will vary by state (and school district). Below is a list of basic questions that a pediatrician should ask of the local schools. Seeing the following table for an example of division of responsibilities.*

1. Is there a licensed professional nurse overseeing health needs of students in the school?
2. What is the ratio of professional nurse time to students served?
3. Who are the staff delivering daily health care tasks?
4. How are the unlicensed assistive personnel trained to deliver health care tasks?

| School Health Aid* | School Nurse* |
| --- | --- |
| - Immediate supervisor is school administrator. - Develops Significant Health Concerns List per school and confidentiality guidelines. - Maintains health office visit logs, health records and immunization records. - Notifies RN regarding questions or concerns. - Notifies Principal and RN in case of serious accident/injury, communicable diseases, immunization status or health related problems. - Assist RN in scheduling, coordination and performing mandated school Hearing/Vision Screening for students. - Administers medications as trained and delegated by RN per district and state guidelines and policy. - Provides specific health interventions to students as trained and delegated by RN. - Provides first aid in accordance with state guidelines. - Maintains health office supplies including AED monitoring. - Provides field trip preparation with RN guidance. - Provides student hearing and vision screening results to RN and/ or special education staff for IEP health assessments. - Models positive health and hygiene behavior (prevention of illness). - Records, contacts and submits documentation to School Medicaid Program for identified students. - Models positive and appropriate communication. | - Maintains licensure by Colorado Board of Nursing and Colorado Department of Education. - Immediate supervisor is building administrator, district health services director, contracted health services program manager, special education coordinator, or other designated personnel. - Determines need for Student Health Plans based on Significant Health Concerns list. - Responds to urgent concerns within 15 minutes. Responds to questions within 24 hours. - Provides appropriate services to student/families/staff with health-related concerns (direct or consultation). - Provides expertise, support and feedback to collaborate on student health needs and concerns that may arise. - Provides consultation and assistance with school Hearing/Vision Screening. - Trains and delegates medication administration to school staff per state and district guidelines, protocols and policies. - Trains and delegates specific health procedures per district and state guidelines and policy. - Participates in educational planning and Special Education assessment/staffing as indicated by health conditions. - Provides liaison with outside agencies including physicians, clinics and health departments. - Serves as primary consultant on programs/policies relating to student health (medication, lice, immunization etc.) and participates on selected task force committees and assignments for the District as needed. |

*Based on Jeffco Department of Health Services Clinic Aide Manual*

**Responsibilities based on Colorado-specific laws.*
