# Supplementary material for: School Health: Pediatric Primary Care Curriculum
Source: MedEdPORTAL. 2018 Oct 19;14:10764. doi: 10.15766/mep_2374-8265.10764 (PMC6346276; doi:10.15766/mep_2374-8265.10764)
Supplement: Supplementary file 1 — A. School Health Curriculum Preparation Checklist.docx B. Part 1 Lession Plan.docx C. School Health Didactic Series Presurvey.docx D. School Accommodations Pre Posttest.docx E. Comparison Table.docx F. Part 2 Lesson Plan.docx G. Role-Play.docx H. Part 3 Lesson Plan.docx I. School Personnel Pre Posttest Answer Key.docx J. Responsibilities of School Health Aide and School Nurse.docx K. Medication Administration Form Instructions.docx L. Assignments.docx M. Follow-up Session.docx N. School Health Didactic Series Postsurvey.docx [file mep-14-10764-s001.zip › K._Medication_Administration_Form_Instructions.docx]

**MEDICATION ADMINISTRATION FORM INSTRUCTIONS**

*Please consider that in most schools an unlicensed assistant is accepting and logging student medications and medication forms. Incomplete information may not be caught until it is too late, when the student needs the medication.*

**Forms must include the following information:**

- Child’s full name
- Medication name – use generic names, in case parent brings different brands to school throughout the school year
- Dosage – use milligram (mg) dose rather than volume of medication, in case patient brings different formulations throughout the school year.
- Time – be specific as possible
  - For PRN medications, include how frequently then can be dose (e.g. Q4H PRN)
  - For QD and TID medications, include exact times of school doses (as patient will also be getting doses at home)
- Route – be specific (i.e. oral, eye, ear, rectal, intranasal, topical, inhaled, neb, etc.)
- Specific Instructions
  - Indicate if a patient is developmentally appropriate to carry medication in their backpack rather than leaving medication with the school nurse. This primarily pertains to asthma and anaphylaxis medications.
  - State if medication should be given with food, after eating, etc.
- Purpose – state in patient-friendly language why the medication is prescribed
- Side Effects – state in patient-friendly language the most common side effects and what needs to be reported to the school nurse or parents
- Parent signature and date – without this, the form is invalid
- Health Care Provider signature and date
  - Include printed name and office information in case the school needs to get in contact with you.

**NOTE:** Every school district is different. Please verify with local schools the rules regarding administration and transportation of medications.
