# Supplementary material for: School Health: Pediatric Primary Care Curriculum
Source: MedEdPORTAL. 2018 Oct 19;14:10764. doi: 10.15766/mep_2374-8265.10764 (PMC6346276; doi:10.15766/mep_2374-8265.10764)
Supplement: Supplementary file 1 — A. School Health Curriculum Preparation Checklist.docx B. Part 1 Lession Plan.docx C. School Health Didactic Series Presurvey.docx D. School Accommodations Pre Posttest.docx E. Comparison Table.docx F. Part 2 Lesson Plan.docx G. Role-Play.docx H. Part 3 Lesson Plan.docx I. School Personnel Pre Posttest Answer Key.docx J. Responsibilities of School Health Aide and School Nurse.docx K. Medication Administration Form Instructions.docx L. Assignments.docx M. Follow-up Session.docx N. School Health Didactic Series Postsurvey.docx [file mep-14-10764-s001.zip › L._Assignments.docx]

Follow up Assignments

Thanks for participating in our school health curriculum. We hope that you learned a lot!

There is a follow up session arranged for you in the next 6-8 weeks. Prior to your follow-up session, please complete the following assignments. **You may email electronic copies or bring hard copies to the follow-up session.**

Assignments:

1. **One completed medication administration form from clinic (de-identified).**  *You may use the form designated by your clinic or brought in by a patient.*

1. **A "one liner" of a clinical dilemma (large or small) from your continuity clinic where you had to address a school health-related need for a patient. Please reflect on how the school health session(s) impacted your interaction.** *School health could include bullying, obtaining appropriate school accommodations, diagnosing learning disabilities, etc. We will discuss these dilemmas as a group during the follow-up session.*
