# Supplementary material for: School Health: Pediatric Primary Care Curriculum
Source: MedEdPORTAL. 2018 Oct 19;14:10764. doi: 10.15766/mep_2374-8265.10764 (PMC6346276; doi:10.15766/mep_2374-8265.10764)
Supplement: Supplementary file 1 — A. School Health Curriculum Preparation Checklist.docx B. Part 1 Lession Plan.docx C. School Health Didactic Series Presurvey.docx D. School Accommodations Pre Posttest.docx E. Comparison Table.docx F. Part 2 Lesson Plan.docx G. Role-Play.docx H. Part 3 Lesson Plan.docx I. School Personnel Pre Posttest Answer Key.docx J. Responsibilities of School Health Aide and School Nurse.docx K. Medication Administration Form Instructions.docx L. Assignments.docx M. Follow-up Session.docx N. School Health Didactic Series Postsurvey.docx [file mep-14-10764-s001.zip › M._Follow-up_Session.docx]

**School Health Curriculum**

**Follow-up Session**

**(1 hour)**

**Learning Objectives**

1. Develop an individualized health care plan for a patient with a chronic illness through collaboration with the school nurse, as assessed by a medication administration form assignment.

**Materials/Personnel**

- Facilitators: faculty/resident curriculum leaders
- School Health Didactic Series Post-Survey (*Appendix N*)
- Completed resident assignments (*Appendix L*)
- Medication Administration Form Instructions (*Appendix K*)

**Reflection (10 minutes)**

1. Complete post-didactic series survey (*Appendix N*).
2. Go around the room and ask each resident to name one important take-away from the didactic session(s).
3. Question for the group: How has clinic been since the session(s)? Have you noticed a change in your practice?

**Medication Administration Forms (5 minutes)**

1. Peer “grade” medication administration forms
   1. Resident pairs
   2. Assess completeness and clarity of written communication and provide feedback to partner, based on the Medication Administration Form Instructions (*Appendix K*)
2. Turn in graded medication administration forms

**Clinical Dilemma “One-liners” (30 minutes)**

1. Choose 3-4 volunteers to share their one-liners
2. As a large group, discuss how to tackle each specific dilemma, with a focus on the resources available to the resident in his/her continuity clinic.
3. Remind residents of online resources available to providers and parents of children with special needs (*see References below*).

**Feedback** **(20 minutes)**

1. What was useful about this curriculum? What was not useful?
2. What can we do to make the session(s) more useful to your residency experience?
3. Is there cross-over and/or redundancy with the behavior & development curriculum?
4. What additional questions about school health do you wish we had covered?
5. What did you think about the assignments?

**References – Online Family Resources**

- Colorado Department of Education: <https://www.cde.state.co.us/>
- PEAK Parent Center: <https://www.peakparent.org/>
- Wright’s Law: <http://www.wrightslaw.com/>
- Center for Parent Information and Resources: <http://www.parentcenterhub.org/>
