# Supplementary material for: School Health: Pediatric Primary Care Curriculum
Source: MedEdPORTAL. 2018 Oct 19;14:10764. doi: 10.15766/mep_2374-8265.10764 (PMC6346276; doi:10.15766/mep_2374-8265.10764)
Supplement: Supplementary file 1 — A. School Health Curriculum Preparation Checklist.docx B. Part 1 Lession Plan.docx C. School Health Didactic Series Presurvey.docx D. School Accommodations Pre Posttest.docx E. Comparison Table.docx F. Part 2 Lesson Plan.docx G. Role-Play.docx H. Part 3 Lesson Plan.docx I. School Personnel Pre Posttest Answer Key.docx J. Responsibilities of School Health Aide and School Nurse.docx K. Medication Administration Form Instructions.docx L. Assignments.docx M. Follow-up Session.docx N. School Health Didactic Series Postsurvey.docx [file mep-14-10764-s001.zip › N._School_Health_Didactic_Series_Postsurvey.docx]

School Health Didactic Series Post-Survey

*Please write the last 4 digits of your phone number in the top right corner of every page to help us track results. We promise we won’t look you up! Thank you for your time and thoughtful responses. Your input will help us make this didactic series better.*

How confident are you that you have the knowledge to help a family of child with special health needs obtain necessary school services?

1. Not at all
2. Slightly
3. Somewhat
4. Very

How comfortable do you feel in explaining to a family the accommodations that may be available to their child and how to pursue those accommodations at school?

1. Not at all
2. Slightly
3. Somewhat
4. Very

How comfortable do you feel in contacting a school on behalf of a patient?

1. Not at all
2. Slightly
3. Somewhat
4. Very

Do you have any additional comments about how we could improve this teaching series for the future?
