# Supplementary material for: School Health: Pediatric Primary Care Curriculum
Source: MedEdPORTAL. 2018 Oct 19;14:10764. doi: 10.15766/mep_2374-8265.10764 (PMC6346276; doi:10.15766/mep_2374-8265.10764)
Supplement: Supplementary file 1 — A. School Health Curriculum Preparation Checklist.docx B. Part 1 Lession Plan.docx C. School Health Didactic Series Presurvey.docx D. School Accommodations Pre Posttest.docx E. Comparison Table.docx F. Part 2 Lesson Plan.docx G. Role-Play.docx H. Part 3 Lesson Plan.docx I. School Personnel Pre Posttest Answer Key.docx J. Responsibilities of School Health Aide and School Nurse.docx K. Medication Administration Form Instructions.docx L. Assignments.docx M. Follow-up Session.docx N. School Health Didactic Series Postsurvey.docx [file mep-14-10764-s001.zip › _Educational_Summary_Report_10764.pdf]

MedEdPORTAL®
The Journal of Teaching and Learning Resources

Original Publication

OPEN ACCESS

School Health: Pediatric Primary Care Curriculum

Lauren R. Anderson, MD*, Cheryl Yang, MD, Jillian Mayer Cotter, MD, Christina Baker, RN, Pamela Brunner Nii, RN, Colette Christen, Mandy Allison, MD, MPH, Daniel Nicklas, MD
*Corresponding author: Lauren.anderson2@childrenscolorado.org

Citation: Anderson LR, Yang C, Cotter JM, et al. School health: pediatric primary care curriculum. *MedEdPORTAL*. 2018;14:10764. https://doi.org/10.15766/mep_2374-8265.10764

Copyright: © 2018 Anderson et al. This is an open-access article distributed under the terms of the Creative Commons Attribution license.

Appendices

A. School Health Curriculum Preparation Checklist.docx
B. Part 1 Lession Plan.docx
C. School Health Didactic Series Presurvey.docx
D. School Accommodations Pre Posttest.docx
E. Comparison Table.docx
F. Part 2 Lesson Plan.docx
G. Role-Play.docx
H. Part 3 Lesson Plan.docx
I. School Personnel Pre Posttest Answer Key.docx
J. Responsibilities of School Health Aide and School Nurse.docx
K. Medication Administration Form Instructions.docx
L. Assignments.docx
M. Follow-up Session.docx
N. School Health Didactic Series Postsurvey.docx

All appendices are peer reviewed as integral parts of the Original Publication.

Abstract

Introduction: Pediatric residents encounter issues related to school health (SH) throughout their training, particularly in their continuity clinics, and often serve as liaisons between a patient's medical home and school environment. However, there is currently a paucity of formal education on SH for pediatric residents to prepare them for this role. **Methods:** We created a 3-hour interactive learning conference that was delivered to four groups of six to eight pediatric residents during their intern year by a multidisciplinary team. Our curriculum focused on understanding the differences between individualized educational plans (IEPs), individualized health plans (IHPs), and 504 plans; the IEP process; and communication with schools. Residents were given pre- and postdidactic surveys, completed reflective writings, and participated in feedback sessions. **Results:** Twenty-seven pediatric interns completed the curriculum; 85% and 74% had improved knowledge of IEP/504/IHP and SH personnel, respectively. Eighty-five percent reported feeling comfortable with family conversations about accommodations postcurriculum versus 0% precurriculum. The majority of interns found the curriculum to be valuable to their clinic performance. **Discussion:** Our curriculum offers a unique multidisciplinary approach to teaching and can be easily integrated into other residency programs, even with limited protected didactic time.

Keywords

School Health, Pediatric Residency Curriculum

Educational Objectives

By the end of this activity, learners will be able to:

1. Articulate the differences between an individualized educational plan (IEP), individualized health plan (IHP), and 504 plan, as assessed by a pre- and postdidactic survey.
2. Identify clinical scenarios in which a patient would qualify for an IEP, IHP, and/or 504 plan, as assessed by a pre- and postdidactic survey.
3. Explain an IEP, IHP, or 504 plan to a family so that the family feels confident in advocating for its children's needs to optimize their education within schools, as assessed by observed role-plays.
4. Explain to a patient's family how to request an evaluation for an IEP so the family feels empowered to advocate for its child's school needs, as assessed by graded role-plays.
5. Identify the medical services that can and cannot be provided by the school nurse and other school personnel, as assessed by a pre- and postdidactic survey.
6. Develop an individualized health care plan for a patient with a chronic illness through collaboration with the school nurse, as assessed by a medication administration form assignment.

Introduction

School health (SH) refers to the physical, cognitive, and emotional needs of a child that impact classroom performance and the child's overall development.[1] Given that children spend the majority of their day in school, partnership between pediatric providers and schools is necessary to meet the needs of a growing population of children with chronic disease. According to a statement made by the American Academy of Pediatrics (AAP), "education in school health should be an important part of graduate medical education

for pediatric residents and of continuing medical education for practicing pediatricians."[2] By engaging in an SH curriculum, residents gain skills to effectively discuss school-related topics with families, have increased awareness of school-related challenges that may arise for patients in clinic or at hospital discharge, and develop greater familiarity with the community in which they practice. Additionally, one study demonstrated that pediatricians who received formal SH training were more likely to participate in SH throughout their career than colleagues without training.[3]

Despite this growing need, national surveys indicate that only about 20%-30% of pediatricians have received formal training in residency, leaving a major educational gap.[3-5] One study surveying young pediatricians showed that 22.4% were exposed to SH during their residencies; however, 65.6% are now providing SH services.[6] A needs assessment via surveys of the residents and faculty at our institution demonstrated a similar need.[7] Based on our review of the literature, there is only one prior study evaluating outcomes of a formal SH curriculum that was implemented at a single institution in 1997-1999.[8] This study demonstrated improved self-perceived resident knowledge and skills, but no change in attitudes towards SH. The AAP's Council on School Health recently published the seventh edition of *School Health: Policy and Practice,*[9] a resource for providers interested in SH. Based on our review of the literature in PubMed and *MedEdPORTAL,* there are currently no structured required curricula at other residency programs that focus on SH.

We created and implemented a curriculum that utilizes an interactive and multidisciplinary format to address the educational gap on SH. We chose this format based on adult learning theory, which holds that engagement of an adult learner is important for meaningful retention of knowledge and skills.[10] Additionally, there are many studies that demonstrate the importance of an interactive and multidisciplinary format. One study surveyed 50 pediatric residents from Duke University and found that the majority preferred an interactive learning model.[11] Another study detailing a breastfeeding curriculum for both residents and medical students reported that one of the key components of the curriculum's success was its multidisciplinary approach, which promoted collaboration amongst different specialists.[12] An article on models for pediatric community health education published in *Pediatrics* emphasized the importance of incorporating not only faculty advisors but also interdisciplinary colleagues and community partners.[13] In order to create and deliver an engaging and relevant curriculum, we used an active learning format and incorporated experts from different disciplines.

Our curriculum has six educational goals that target resident knowledge and behaviors. Deliberate SH education aligns with the Entrustable Professional Activity to "provide a medical home for patients with complex, chronic, or special health care needs," which, per the AAP Committee on School Health, includes advocating for patients in the school setting and promoting communication among school authorities and health care providers.[14] Curricular components include knowledge of community resources, interprofessional collaboration, and skills to empower patients and their families to be self-advocates.

Methods

We developed this curriculum as a resident scholarly project at the University of Colorado. Our target learners were first-year pediatric residents, all of whom practiced in primary care settings. After performing a literature review and discussing our plans with a national expert in the field, we completed an institution-wide SH needs assessment via an electronic survey of current pediatric residents and faculty.[7] We designed the curriculum content based on these results, along with input from primary care and behavior and development physicians, as well as school nurses involved in the Children's Hospital Colorado School Health Program. The short-term goal of our work was to increase knowledge of SH as it pertained to accommodations and school personnel. Our ultimate goal was to increase the frequency and effectiveness of resident communication with clinic patients regarding SH needs.

We designed the SH curriculum to be delivered as a single 3-hour educational conference utilizing interactive small-group lectures and role-play, followed by a feedback session. Each educational conference was divided into three parts, which could easily be delivered as separate lectures (Table). Based on space and time available, we repeated the curriculum four times throughout the year. Interns completed the curriculum during a 3-month continuous block that included didactics and clinical experiences in primary care, behavior and development, and emergency medicine. Seven to eight interns participated in each block. We created a curriculum checklist (Appendix A) to improve our efficiency in preparing for subsequent conferences.

Table. School Health Curriculum Sessions and Associated Appendices

Session	Associated Appendices	Duration (Hours)
Part 1: School Accommodations	B, C, D, E	1
Part 2: IEP Process	F, G	1
Part 3: School Staff and Communication	H, I, J, K, L	1
Follow-up session	K, M, N	1

Abbreviation: IEP, individualized educational plan.

For Part 1: School Accommodations, our goal was to introduce learners to three commonly used accommodation plans: the individualized education plan (IEP), individualized health plan (IHP), and 504 plan. A lesson plan with detailed teaching points was provided (Appendix B). This session was led by senior residents and primary care faculty members. We administered learner assessments (Appendices C & D) prior to the didactic to assess previous exposure to SH and baseline knowledge of school accommodations. We then discussed definitions and applications of the three plans. A comparison table (Appendix E) was utilized to highlight differences in these plans, and sample plans from the internet were reviewed. We deliberately avoided a full dissection of an IEP, which was covered in the residency's behavior and development curriculum. At the end of Part 1, we repeated the SH accommodations assessment form.

For Part 2: IEP Process, our goal was to provide participants with a clear understanding of how students obtain IEPs so participants would be able to explain these steps to a family. A lesson plan with detailed teaching points was provided (Appendix F). This session was facilitated by a family navigator, a registered nurse helping clinic families access community resources. We discussed the time line for obtaining an IEP, the legal obligation of schools, and common challenges. Parents of patients with special needs and, on occasion, older patients themselves then joined the group to share their experiences with IEPs and to field questions from the residents. Residents then divided into small groups to work through two role-play scenarios (Appendix G) to practice explaining the IEP process. The family navigator and family members offered feedback after each role-play, based on a provided rubric.

For Part 3: School Staff and Communication, our goal was to introduce learners to the roles of SH personnel and how to communicate patient needs to a school. A lesson plan with detailed teaching points was provided (Appendix H). School nurses facilitated the session. We administered a learner assessment (Appendix I) to assess baseline knowledge of school personnel. We then discussed staffing and scope of practice of SH providers, with use of a comparison table highlighting differences between the two most common provider types: school nurses and unlicensed assistive personnel (Appendix J). Best practices for communicating with schools were addressed, and practical tips for completing medication administration forms (Appendix K) were shared. We dedicated the remainder of the time to a question-and-answer session with the school nurses. We also repeated the school personnel assessment. At the end, we distributed postdidactic assignments (Appendix L) to be completed by the time of the follow-up session.

The follow-up session was scheduled 6-8 weeks after the educational conference. A lesson plan was provided (Appendix M). The session was facilitated by a senior resident who was not involved in the learning conference. We administered a learner assessment (Appendix N) to measure resident SH attitudes. Residents were asked to share how the knowledge they had gained in the curriculum impacted

their clinical experiences and to offer advice for curriculum improvement. We reviewed their assignments and facilitated peer grading of their completed medical administration forms based on inclusion of key information detailed in Appendix K. Residents shared and discussed clinical SH dilemmas.

In summary, our SH curriculum involved 4 hours for interactive lecture and reflection utilizing review of real accommodation plans, question-and-answer sessions with parent(s) and/or children currently receiving accommodations in school, and small-group activities. The curriculum was first delivered in August-September 2016. To assess the curriculum's effectiveness, we administered pre- and postdidactic surveys to assess SH knowledge and behavior via multiple-choice, Likert-scale, and free-response questions. We did not reassess behavior in the postdidactic survey as we felt the time interval between surveys was too short to measure sustained change. We trialed the survey on a small cohort and revised based on feedback prior to distribution. We analyzed mean differences between pre- and postdidactic scores and gathered qualitative data from written and verbal learner comments.

Results

We provided our curriculum to 27 of 32 (84%) pediatric interns during the 2016-2017 academic year. Five interns did not receive the curriculum due to scheduling conflicts. Those interns had access to all of the learning materials via Brightspace, our learning content management system, and attended the feedback session.

We administered pre- and postdidactic surveys to all 27 interns present for the 3-hour educational conference. Surveys were not provided to those who accessed the materials online. Forty-one percent of interns reported some SH experience prior to the session, including "discussion with an attending or health care team member" or a "half day of clinic at a school-based health center"; the rest had no experience. Eighty-five percent and 75% of the interns had improved knowledge scores for IEP/504/IHP and SH personnel, respectively (Figure 1). Few interns reported being somewhat or very comfortable with family conversations about accommodations and school communication precurriculum (0% and 11%, respectively) versus postcurriculum (96% and 85%, respectively; Figure 2 and Figure 3).

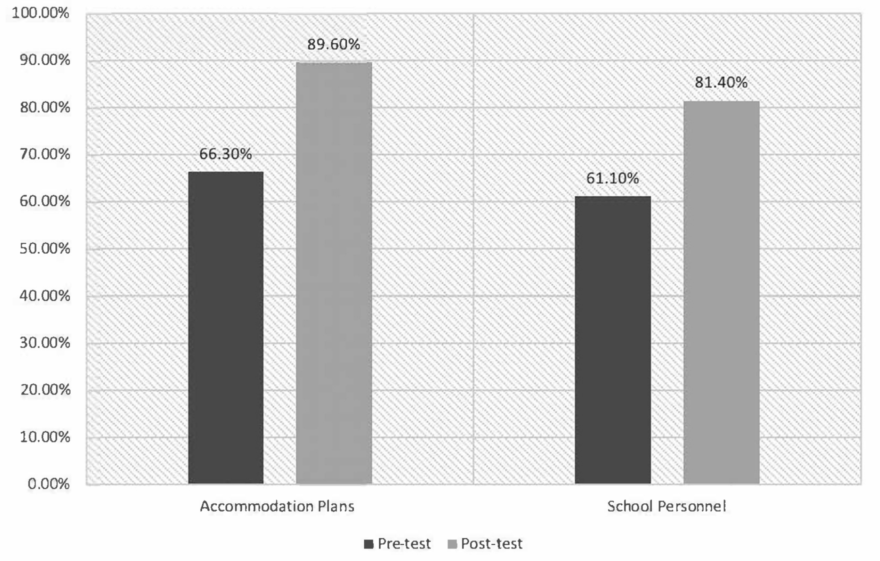

Figure 1. Mean school health (SH) knowledge score: comparison of pretest versus posttest results. Test scores were calculated as percentage correct out of 100%. With SH education, residents showed improvement in knowledge with respect to both accommodation plans and school personnel.

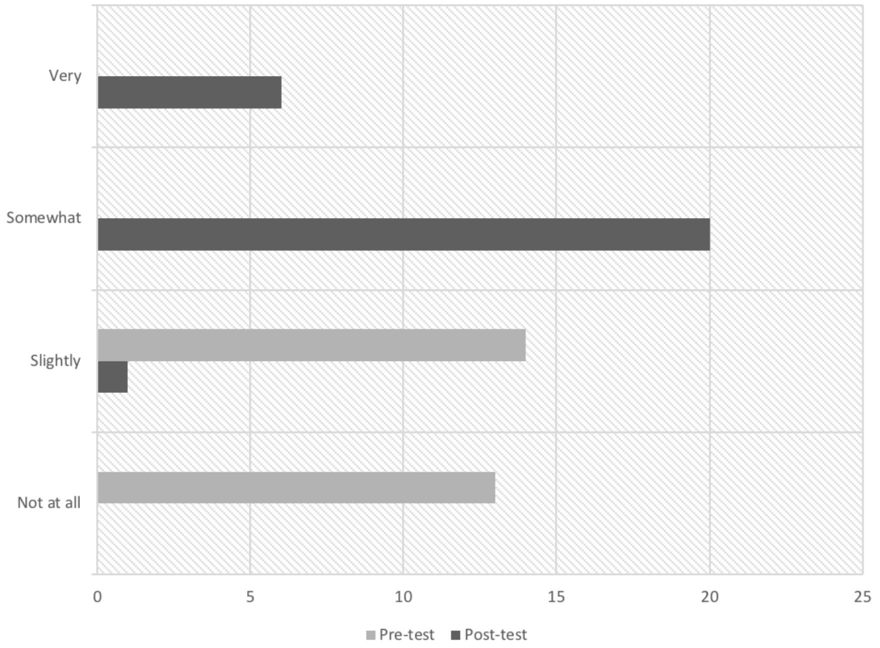

Figure 2. Resident comfort with explaining accommodations to families: pretest versus posttest. School health (SH) education improved residents' comfort with explaining accommodations to families. Most residents were initially slightly or not at all comfortable, but after SH education, most felt somewhat or very comfortable.

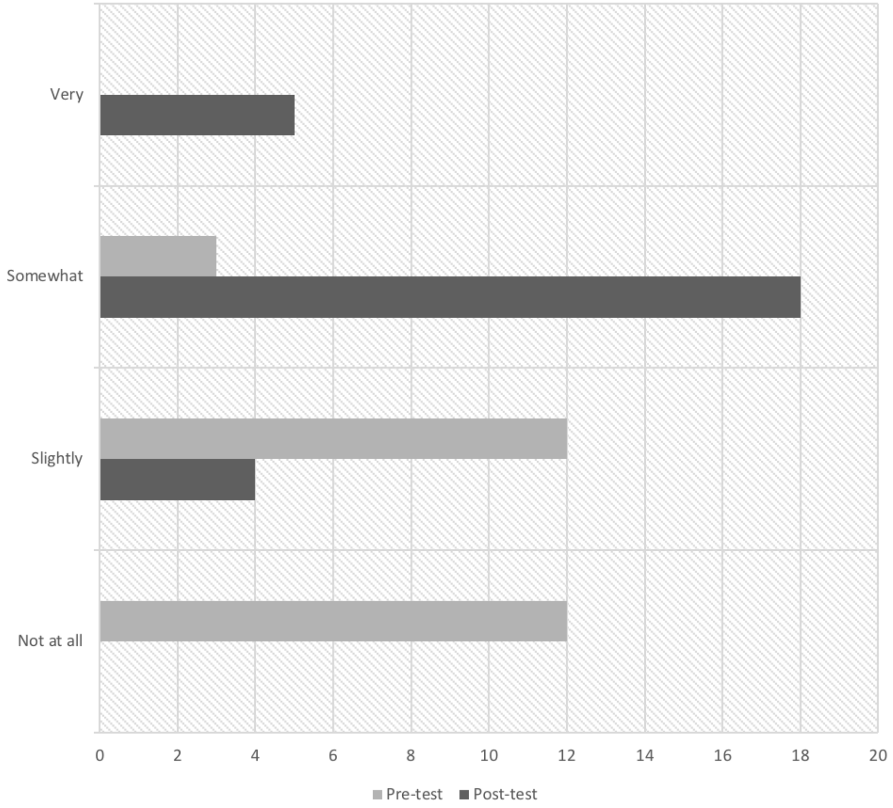

Figure 3. Resident comfort with contacting a school on behalf of a patient: pretest versus posttest. School health (SH) education improved residents' comfort with contacting a school on behalf of a patient. Most residents were initially slightly or not at all comfortable, but after SH education, most felt somewhat or very comfortable.

During feedback sessions, we identified representative themes, which included (1) increased awareness of how a child's medical diagnosis may affect him/her in school, (2) increased discussions with families of children who are struggling in school to help facilitate obtaining appropriate accommodations, and (3) increased communication with schools via written medical forms and letters. Interns recommended less role-play, increased time for questions and answers, and coverage of additional SH topics, including bullying. The select interns who did not attend the educational conference had more difficulty reflecting on clinical experiences and did not endorse changes in their knowledge or comfort of SH topics.

The postsurveys and feedback discussions suggested that interns found the curriculum to be highly valuable to their clinic performance. Comments of praise included the following:

- "Very valuable; explaining IEP, 504 and IHP was very helpful and empowering."
- "Appreciated dedicated time to explaining IEP, 504, IHP and examples of where each are appropriate. Valued [the family navigator's] contributions including website [recommendations] & examples of how to advocate for families."
- "I liked that there was specific emphasis on practicing the implementation of our action plans. Also, loved the patients and parent visits."

Discussion

Traditionally, exposure to SH in residency has been limited and informal. Our curriculum ensures that pediatric residents receive standardized training that equips them with a toolkit to address the school concerns that inevitably surface in their primary care clinics. Additionally, our program offers residents their only formal opportunity in training to meet with key members of the complex patient care team: family navigators, school nurses, and patient families.

Our results show improved resident competency and comfort with SH topics, as well as increased appreciation for the multidisciplinary approach to care. Knowledge scores improved more dramatically for school accommodations than for school communication, which likely reflects the uneven distribution of time (2:1) spent covering these topics. Arguably, improved understanding of school systems and staff is more important because it is applicable to the majority of resident clinic patients compared to the smaller percentage requiring formal accommodations. Additional time dedicated to school communication may be merited and could include incorporation of more school staff facilitators, such as unlicensed assistive personnel, or an additional session to review real-life cases and write IHPs. Notably, our assessment tools only evaluated change in knowledge and comfort immediately following participation in the curriculum and may not reflect long-term change. Repeat assessments 6 months to 1 year later were outside the scope of our study but could provide insight on how to modify the lesson plans and allocation of time.

We anticipate that increased SH awareness among our residents will translate to improved patient care, in which families are better supported in the process of obtaining school resources and communication between physicians and schools is more effective. Our project only measured change in residents' self-reported attitudes. We hope that future research can elucidate the impact of this curriculum on residents' behaviors, which could be assessed via an objective structured clinical examination or patient surveys.

This curriculum was designed to allow for easy implementation at other pediatric residency programs and across disciplines. We feel that structured SH training would benefit nursing, social work, and advanced care provider students, in addition to physicians. While our block schedule allowed us to deliver the didactic portion as a single educational conference, the curricular content can easily be separated into three distinct 1-hour sessions and offered as part of a morning or noon conference series. In the case of large-group teaching, we would recommend increasing facilitator presence to allow for small breakout groups of six to 10 learners, which we feel enhanced our participation and discussion, particularly during

the question-and-answer sessions. We utilized family navigators who were familiar with school accommodations; a social worker or provider knowledgeable in SH could act as a substitute. The interns appreciated the participation of school nurses, who could be substituted for by school administrators or a provider knowledgeable about local school systems. Patient family involvement was key, but a spokesperson from a local family support group or a special education teacher who can provide personal anecdotes could act as a substitute. We utilized local examples of school accommodations and medication administration forms, which may have slight differences from those used in other regions of the country. However, locally relevant samples could be identified by a developmental pediatric or primary care clinic, and more generic materials are available online through U.S. Department of Education and parent support organizations, links to which we have included in our lesson plans.

In reflecting on the implementation of our curriculum, we see that time was the biggest challenge. Significant organization was required to coordinate a large number of facilitators over four iterations of the curriculum. Utilization of the checklist streamlined this process. Identifying committed facilitators willing to return for all sessions was critical and has proven important for sustainability. Procuring protected educational time for all trainees was the largest limiting factor. Due to our program's scheduling constraints, some interns did not receive the curriculum until their ninth month of intern year and felt disadvantaged by having received the material so late. Five interns missed the didactics entirely and did not feel that the electronic teaching materials were adequate. Online modules or videos, as well as relocating the didactics to intern orientation prior to the start of the academic year, could improve access to and timeliness of the curriculum. As highlighted in feedback, our 3-hour single-conference format restricted the length of the question-and-answer sessions, which ideally would be allocated a separate didactic block, and forced us to skip over some school-related topics that interns felt were high yield. While many of these topics, such as bullying, are covered in our primary care curriculum during continuity clinic and clinic block rotations, recurrent comments reflected a need to better integrate our SH training with the broader primary care teaching.

We have continued to provide our curriculum during the 2017-2018 academic year, with plans to continue in 2018-2019 under new resident leadership. We are now looking to create more accessible and effective online resources via multimedia modules or podcasts.

Lauren R. Anderson, MD: Clinical Instructor, Department of Pediatrics, University of Colorado School of Medicine

Cheryl Yang, MD: Clinical Instructor, Department of Pediatrics, University of Colorado School of Medicine

Jillian Mayer Cotter, MD: Pediatric Hospital Medicine Fellow, Department of Pediatrics, University of Colorado School of Medicine

Christina Baker, RN: Clinical Nursing Informaticist, Children's Hospital Colorado; School Health Consultant, Children's Hospital Colorado

Pamela Brunner Nii, RN: Clinical Nurse, School Health Program, Children's Hospital Colorado

Colette Christen: Family Navigator, Neuroscience Institute, Children's Hospital Colorado

Mandy Allison, MD, MPH: Associate Professor, Department of Pediatrics, University of Colorado School of Medicine

Daniel Nicklas, MD: Assistant Professor, Department of Pediatrics, University of Colorado School of Medicine

Disclosures
None to report.

Funding/Support
None to report.

Informed Consent
All identifiable persons in this resource have granted their permission.

Prior Presentations
Mayer J, Anderson L, Yang C, Seltz L. School health in residency: identifying gaps in pediatric training. Abstract presented at: Association of Pediatric Program Directors Spring Meeting; April 2016; New Orleans, LA.

Yang C, Anderson L, Cotter JM, Seltz LB. School health in residency: identifying gaps in pediatric training. Abstract presented at: Pediatric Academic Societies Meeting; May 2016; Baltimore, MD.

Anderson L, Yang C, Cotter J, Nicklas D. Incorporating school health into residency education. Work in progress presented at: Academic Pediatrics Association Region IX/X Meeting; February 2017; Monterey, CA.

Yang C, Anderson L, Cotter J, Allison M, Nicklas D. Creation of a school health curriculum. Abstract presented at: Association of Pediatric Program Directors Spring Meeting; April 2017; Anaheim, CA.

Yang C, Anderson L, Cotter J, Allison M, Nicklas D. Creation of a school health curriculum. Abstract presented at: Pediatric Academic Societies Meeting; May 2017; San Francisco, CA.

Ethical Approval
Reported as not applicable.

References

1. Bradford BJ. Resident education in school health: an issue whose time has come. *Pediatrics*. 1993;92(3):496-498.
2. Section on School Health. Residency training and continuing medical education in school health.*Pediatrics*. 1993;92(3):495-496.
3. Nader PR, Broyles SL, Brennan J, Taras H. Two national surveys on pediatric training and activities in school health: 1991 and 2001. *Pediatrics*. 2003;111(4):730-734.
4. Barnett S, Duncan P, O'Connor KG. Pediatricians' response to demand for school health programming. *Pediatrics*. 1999;103(4):e45. https://doi.org/10.1542/peds.103.4.e45
5. Bradford BJ. School health in pediatric residency training: 1994. *Arch Pediatr Adolesc Med*. 1996;150(3):315-318. https://doi.org/10.1001/archpedi.1996.02170280085016
6. Chilton LA. School health experience before and after completion of pediatric training. *Pediatrics*. 1979;63(4):565-568.
7. Yang C, Anderson L, Cotter JM, Seltz LB. School health in residency: identifying gaps in pediatric training. Abstract presented at: Pediatric Academic Societies Meeting; May 2016; Baltimore, MD.
8. Starr S, Perrin EC. Teaching about schools during pediatric residency. *Ambul Pediatr*. 2005;5(3):178-184. https://doi.org/10.1367/A03-020R1.1
9. Gereige RS, Zenni EA, eds. *School Health: Policy and Practice*. 7th ed. Elk Grove Village, IL: American Academy of Pediatrics; 2016.
10. Knowles MS. *The Adult Learner: A Neglected Species*. 4th ed. Houston, TX: Gulf Publishing; 1990.
11. Turner DA, Narayan AP, Whicker SA, Bookman J, McGann KA. Do pediatric residents prefer interactive learning? Educational challenges in the duty hours era. *Med Teach*. 2011;33(6):494-496. https://doi.org/10.3109/0142159X.2010.542524
12. Ogburn T, Espey E, Leeman L, Alvarez K. A breastfeeding curriculum for residents and medical students: a multidisciplinary approach. *J Hum Lact*. 2005;21(4):458-464. https://doi.org/10.1177/0890334405280990
13. Shipley LJ, Stelzner SM, Zenni EA, et al. Teaching community pediatrics to pediatric residents: strategic approaches and successful models for education in community health and child advocacy. *Pediatrics*. 2005;115(suppl 3):1150-1157. https://doi.org/10.1542/peds.2004-2825J
14. Committee on School Health. School health assessments. *Pediatrics*. 1991;88(3):649-651.

Received: May 20, 2018 | **Accepted:** September 16, 2018 | **Published:** October 19, 2018
